# Measurement of Retinal Changes in Primary Acute Angle Closure Glaucoma under Different Durations of Symptoms

**DOI:** 10.1155/2019/5409837

**Published:** 2019-12-04

**Authors:** Xiaolu Zhu, Wen Zeng, Shengyu Wu, Xiaomin Chen, Tian Zheng, Min Ke

**Affiliations:** Department of Ophthalmology, Zhongnan Hospital of Wuhan University, Wuhan, China

## Abstract

**Purpose:**

To investigate the changes of retinal nerve fiber layer (RNFL) in patients after an attack of primary acute angle closure glaucoma (PAACG) and to assess the impact of attack time on prognosis of retinal changes.

**Design:**

cross-sectional study.

**Methods:**

Twenty-six patients with unilateral PAACG attack and cataracts from 2017 to 2019 were enrolled. Eyes with PAACG attack time less than 1 day constituted the group A (*n* = 13), while eyes with PAACG attack time more than 1 day constituted the group B (*n* = 13). All patients received phacoemulsification and viscogoniosynechialysis after intraocular pressure (IOP) lowering. All patients underwent ophthalmic examinations including IOP, best-corrected visual acuity (BCVA), and visual field (VF). Optical coherence tomography angiography (OCTA) was used to obtain circumpapillary RNFL vessel density (cpVD). Spectral domain optical coherence tomography (SD-OCT) was used to examine the peripapillary RNFL and macular ganglion cell complex (GCC). All patients accepted 2 assessments before and 1 month after the procedure.

**Results:**

The IOP of all patients recovered to normal (12.77 ± 2.65 mm Hgvs. 12.77 ± 3.85 mmHg, *p*=0.834) after the procedure. Patients in the group A had better BCVA improvement than those in the group B (1.32 ± 0.84 vs. 0.50 ± 0.21, *p*=0.004), as well as better mean defect (MD) values from VF (−3.65 ± 2.54 vs −16.05 ± 5.99, *p* < 0.001). Compared with group B, patients in the group A had thicker macula (Fovea area: 255.00 ± 27.94 *μ*m vs. 203.92 ± 59.73 *μ*m, *p*=0.010), thicker GCC (82.62 ± 8.76 *μ*m vs. 65.23 ± 18.56 *μ*m, *p*=0.005), and thicker RNFL (105.08 ± 9.38 *μ*m vs. 77.69 ± 20.23 *μ*m, *p* < 0.001). Higher blood flow density in all-plexus peripapillary retina was observed in the group A eyes compared with group B (full sector: 0.56 ± 0.02 vs. 0.41 ± 0.07, *p* < 0.001). In both groups, the association between average RNFL thickness and cpVD as well as MD values and pattern standard deviation (PSD) values from VF was stronger (*R*^2^ = 0.58, 0.60, −0.54, respectively, all *p* < 0.001) than the association between GCC thickness and cpVD, as well as MD values and PSD values (*R*^2^ = 0.37, *p*=0.001; *R*^2^ = 0.37, *p*=0.001; *R*^2^ = −0.27, *p*=0.007).

**Conclusion:**

Patients with attack time less than 1 day had better retinal thickness and all-plexus peripapillary retina blood flow density. Controlling the attack time could decrease retinal damage by PAACG.

## 1. Introduction

Primary acute angle closure glaucoma (PAACG), an acute ocular hypertension disease that may lead to irreversible damage to the optic nerve and other ocular tissues, is one of the most common types of glaucoma, especially among Asians [1]. Ocular biometric characteristics of Asians result in higher prevalence in Asia [2]. Shallow anterior chamber depth and lens-related and retrolenticular mechanisms lead to overcrowding anterior structure and decreased opening of trabecular meshwork [3–5]. PAACG begins with acute primary angle closure (APAC) attack and develops to optic neuropathy later [6, 7]. It was reported that high intraocular pressure (IOP) caused vascular insufficiency and ganglion cell apoptosis, leading to optic neuropathy and vision loss [6]. Recent reports also stated that PAACG-induced retinal nerve fiber layer (RNFL) thickness change was an evolving process between 2 and 16 weeks after the attack [8]. At present, the related examinations for glaucoma diagnosis and condition monitoring included visual acuity (VA), IOP, visual field (VF), and optical coherence tomography (SD-OCT). In addition, optical coherence tomography angiography (OCTA), as a new method, provides a more sensitive analysis on glaucoma diagnosis and mechanism at the vascular levels [9]. Previous studies reported the close relationship between these indicators [10], but the association between the PAACG attack time with the fundus condition is yet to be investigated.

The shallow anterior chamber depth is a common feature of glaucoma patients [11]. PAACG patients benefit a lot from the combined procedure of phacoemulsification and viscogoniosynechialysis, which deal with cataract problem and expand the anterior chamber depth to reduce the patients' suddenly increased IOP at the same time [12]. This study aimed to investigate the structure and vessel changes of the RNFL of PAACG eyes from patients suffering from different attack times. Phacoemulsification and viscogoniosynechialysis were performed after resolution of the acute episode in PAACG patients. In addition, VA, IOP, VF, and their correlations were investigated in our study. Our results indicated that shorter attack time resulted in better visual acuity, better VF, higher vascular density, thicker macula, and thicker RNFL.

## 2. Materials and Methods

### 2.1. Patients

Study subjects were recruited from patients with an episode of unilateral PAACG attack and cataracts at the Division of Glaucoma, Department of Ophthalmology, Zhongnan Hospital of Wuhan University, China, between January 2017 and April 2019. The study protocol was approved by the Institutional Review Board of Zhongnan Hospital of Wuhan University and adhered to the Declaration of Helsinki. The study protocol was registered on http://www.ClinicalTrials.gov (ChiCTR1800017849). Written informed consent was obtained from each patient in this study prior to enrollment. The inclusion criteria for PAACG patients were as follows: (1) IOP > 21 mmHg with ≥2 following symptoms: visual blurring, ocular pain, headache, nausea, and vomiting; (2) the presence of any following signs on silt-lamp examination: conjunctival congestion, a middilated unreactive pupil, corneal edema, and a shallow anterior chamber; (3) a closed angle observed by gonioscopy; (4) duration of PAACG attack < 120 hours; (5) IOP < 21 mmHg after medical therapy and surgery. The exclusion criteria were as follows: (1) bilateral attack; (2) secondary angle closure caused by phacomorphic angle closure, subluxed lens, and neovascularization; (3) a history of vitreoretinal or corneal surgery in the attack eye; (4) refraction > 5.0 *D* (sphere) and/or 3.0 *D* (cylinder); (5) incomplete clinical data, poor-quality OCT scans, the ratio of false negative or false positives in VF examination > 30%, or fixation loss of >20%; (6) Severe corneal edema after IOP control.

### 2.2. Examinations

Patients were divided into 2 groups according to their attack time: <1 day (group A) and ≥1 day (group B). All subjects underwent ophthalmic examinations including best-corrected visual acuity (BCVA, measured by Snellen chart), IOP measurement, slit-lamp examination, fundus examination, and gonioscopy. IOP was measured by iCare tonometry or Goldmann applanation tonometry. Gonioscopy was examined with a Goldmann 2-mirror lens at high magnification (×16). VF testing was performed using 24–2 pattern Swedish interactive threshold algorithm on the Humphrey Field Analyzer (Carl Zeiss, USA). VF indices included mean defect (MD) and pattern standard deviation (PSD). Normal VF was defined as MD and PSD within 95% confidence intervals and a glaucoma hemifield test result “within normal limits.” RNFL images were obtained using the stratus OCT 3000 (Carl Zeiss) with peripapillary RNFL thickness calculated automatically, including average, superior, nasal, inferior, and temporal quadrants. Only high-quality images with 3 ≤ signal strength ≤ 10 were included. Macular thickness in scans with a 512 × 128 mm field and ganglion cell complex (GCC) thickness were also assessed with SD-OCT. Vessel perfusion density was measured within the cpVD in scans with a 6 × 6 mm field centered on the optic nerve head. Vessel density within the superficial retina layer was measured using the ZEISS CIRRUS™ OCT Angiography systemRTVue-XR Avanti.

### 2.3. Procedures

All patients underwent phacoemulsification and viscogoniosynechialysis. Two scheduled ophthalmic examinations were performed after IOP controlling: the first one before the procedure and the second one 1 month after the procedure. The patients with IOP > 21 mmHg 1 day after these procedures were further treated with IOP-lowering drugs, such as (1) beta-blockers, (2) carbonic anhydrase inhibitors, (3) pilocarpine, and (4) adrenergic agonists. Additional clinic visits and investigations were scheduled when required.

### 2.4. Statistical Analysis

All data were analyzed using the SPSS version 20.0. Independent-sample *T* test was used for the significance of differences between IOP, VA, OCT parameters, vessel flow density, and VF indices including MD and PSD. Pearson correlation was used to evaluate single correlation between average RNFL thickness, average GCC thickness, cpVD, and VF indices. The significance was set as a two-sided *p* < 0.05.

## 3. Results

### 3.1. Shorter Attack Time Indicated Better BCVA Improvement

**Figure 1:**
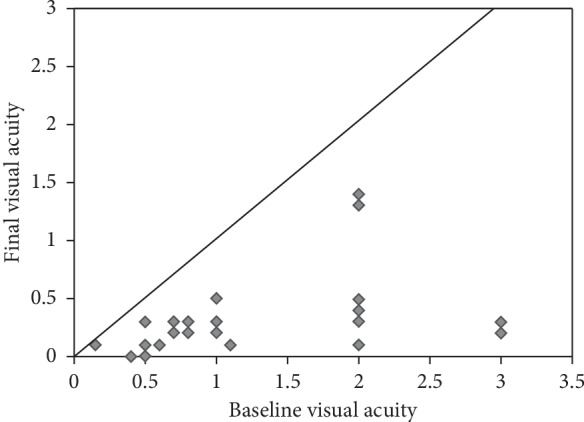
Baseline and final logMAR BCVA of patients in this study. Dots below the line indicate improved visual acuity, and dots above the line indicate worse visual acuity.

A total of 26 eyes from 26 patients with unilateral PAACG were enrolled in this study. The characteristics of all the patients are listed in Table 1. Age (*p*=0.092) and gender (*p*=0.855) were comparable between groups. The mean duration of the PAACG attack was 10.0 ± 6.2 hours (range 2 to 19 h) and 79.3 ± 24.7 hours (range 28 to 120 h), respectively. The mean cpRNFL thickness, baseline vertical cup-to-disc ratio, mean GCC thickness, cpVD, and MD value from VF had no significant differences in the 2 groups (all, *p* > 0.05). The BCVA before and after surgery in both groups is listed in Table 2. The distributions of the initial and final logMAR VA were shown in Figure 1. Compared with the initial mean BCVA, the final mean BCVA was significantly improved in both groups. In addition, the group A (attack time < 1 day) had a better BCVA and greater improvement than the group B (attack time > 1 day).

### 3.2. Patients with Shorter Attack Time Had Better VF

As VF was measured 1 month after surgery, the MD and PSD values were significantly lower in patients from the group A than those from the group B (−3.65 ± 2.54 vs. −16.05 ± 5.99, *p* < 0.001; −2.75 ± 1.10 vs. 9.43 ± 2.93, *p* < 0.001; Table 2).

### 3.3. Patients with Shorter Attack Time Had Thicker Macula, GCC, and RNFL

The fovea and inner circle and outer circle thicknesses of macula were significantly different between groups 1 month after surgery (Table 2). One month after surgery, the macular fovea thickness in the group A was higher than that in group B (255.00 ± 27.94 *μ*m vs. 203.92 ± 59.73 *μ*m, *p*=0.010). The inner circle of macular thickness was higher in the group A (304.38 ± 23.41 *μ*m vs. 254.23 ± 58.13, *p*=0.008). The outer circle of macular thickness was also higher in the group A (269.92 ± 21.68 *μ*m vs. 231.00 ± 32.39 *μ*m, *p*=0.001). These results indicated that the changes of macular thickness were correlated with attack time. Longer attack time indicated thinner macula. The average thickness of GCC was significantly higher in the group A than those in the group B 1 month after surgery (82.62 ± 8.76 *μ*m vs. 65.23 ± 18.56 *μ*m, *p*=0.005).

The superior, temporal, inferior, nasal, and average peripapillary RNFL thickness 1 month after surgery were listed in Table 2. Compared with group B, group A had a significantly thicker RNFL (105.08 ± 9.38 *μ*m vs. 77.69 ± 20.23 *μ*m, *p* < 0.001). Quadrants analyses showed higher superior (137.54 ± 12.23 *μ*m vs. 103.92 ± 27.74 *μ*m, *p*=0.001) and inferior quadrants (149.92 ± 21.73 *μ*m vs. 100.38 ± 24.38 *μ*m, *p* < 0.001) in the group A but comparable thickness in the temporal and nasal quadrants (*p*=0.543; *p*=0.057).

### 3.4. Patients with Shorter Attack Time Had Higher cpVD

The OCTA-derived vessel blow densities from both groups are presented in Table 2. Eyes from the group A had significantly greater cpVD (circumpapillary RNFL vessel density) than those from the group B in the central area (0.34 ± 0.03 vs. 0.29 ± 0.04; *p*=0.002), in the inner area (0.56 ± 0.03 vs. 0.42 ± 0.06; *p* < 0.001), in the outer area (0.56 ± 0.03 vs. 0.40 ± 0.08; *p* < 0.001), and in the full area (0.56 ± 0.02 vs. 0.41 ± 0.07; *p* < 0.001).

### 3.5. Relationship between VF Parameters, Average RNFL Thickness, and cpVD

The correlation coefficients between VF parameters, average RNFL thickness, and cpVD in both groups 1 month after surgery are summarized in Table 3. The significant correlations were found between all these parameters (*r* > 0.4, *p* < 0.05). In both groups, the association between the average RNFL thickness and cpVD, as well as MD values and PSD values from VF, was stronger (*R*^2^ = 0.58, 0.60, −0.54, respectively, all, *p* < 0.001) than the association between the GCC thickness and cpVD, as well as MD values and PSD values (*R*^2^ = 0.37, *p*=0.001; *R*^2^ = 0.37, *p*=0.001; *R*^2^ = −0.27, *p*=0.007).

### 3.6. Patients with Shorter Attack Time Had Better Retinal Function

**Figure 2:**
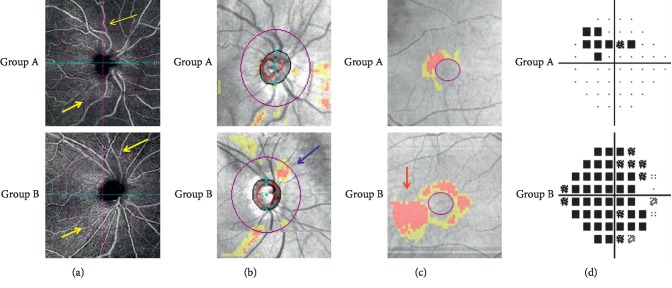
Representative retinal damage images of 2 patients from different groups. (a) cpVD: OCT angiography image of a 6 × 6 mm section, centered on the optic disc head. Group A showed a better blood flow density than group B (yellow arrowheads). (b) ONH: RNFL thickness analysis examined by OCT. Group B was detected a more apparent RNFL defect in the superior region than group A (blue arrowheads). (c) GCC: ganglion cell complex thickness. An apparent GCC defect was detected in the inferotemporal area (red arrowheads). (d) MD: VF pattern deviation plot. Superior visual field loss was detected in group B patient.

The fundus conditions of 2 representative patients from both groups are shown in Figure 2. The visual function of the patient from the group A was better than that from the group B.

## 4. Discussion

In our study, we used the diagnostic ability of BCVA and structural and vascular changes from macula and RNFL to detect retinal changes in PAACG patients. Most patients had good visual outcomes after surgery, and patients with shorter attack time (less than 1 day) got better BCVA. Patients with shorter attack time had thicker macula, GCC, and RNFL. Moreover, the retinal vessel densities in the peripapillary areas were significantly greater in the patients with shorter attack time.

SD-OCT and OCT-A were recently developed to observe the retinal function in glaucoma patients in both the foveal and disc areas, in an accurate, convenient, and noninvasive way [13, 14]. Reduced blood flow in the choroid, retina, and optic nerve head in glaucoma patients was proved using OCT angiography [15–18]. Our results showed that the RNFL thickness was higher in PAACG patients at the early stage of relief. This thickening is commonly due to retinal edema. The results of our studies also demonstrated that the retinal thickness of all PAACG patients slowly decreases with the passage of time, which was consistent with the findings of Rao et al. [19] and Wang et al. [20]. Our results suggested that the speed and extent of retinal function loss were closely proportional to the attack time. These results might be related to 2 classic theories: a mechanical theory and a vascular theory [21, 22]. The mechanical theory noted that increased IOP led to elongation, stretching, and collapse of the laminar beams and retinal ganglion cell (RGC) axons damage [23]. According to the vascular theory, insufficient blood supply on account of either increased IOP or other risk factors reducing ocular blood flow may cause glaucomatous optic neuropathy (GON) [24]. Hayreh et al. also noted that the retinal circulation was not easily damaged at 30 mmHg IOP, unless IOP reached 40–70 mmHg. When IOP increased to 70 mmHg, the choroid capillaries in disc area almost disappeared, resulting in slow retinal circulation. These theories and phenomena may explain why prolonged periods of high IOP have significant effects on the fundus of PAACG patients.

In our studies, the thickness of the retina was significantly reduced on the superior and inferior areas, while the changes of nasal side and temporal side in the RNFL were lighter. These results were consistent with the findings of Igarashi et al. [9], who reported that the superior and inferior areas of the optic disc were more affected by glaucoma, which was the pathological characteristic of glaucomatous optic neuropathy [18].

In this study, we also evaluated the relationship between VF and fundus changes. There was a strong correlation between the VF loss and cpVD (all, *p* < 0.001), suggesting that reduced vessel flow density was associated with more advanced stages of glaucomatous VF damage. Moreover, we also found that changes in RNFL and RGC thickness often occurred later than changes in VF and blood flow density [25, 26]. There are several possible explanations for the independent association of vascular density and the MD of VF: first, it may be due to the reduced and dysfunctional RGCs (i.e., preapoptosis). Blood flow is therefore lower in vascular density and less sensitive to VF. Since these dysfunctional RGCs did not shrink, and the reduction in RNFL thickness and marginal area might not be discovered [10]. In addition, histological studies showed that there was only moderate correlation between RNFL thinning and RGC loss, indicating that RNFL thinning did not fully reflect the RGC function [5, 27]. Thus, a stronger correlation between vessel density and VF impairment indicated that vessel flow density was a better reflection of RGC function than structural loss [28]. In other words, these 2 indicators may play major roles in detecting early stage of glaucoma.

Our research had several limitations that need to be considered. All patients were selected from a single institution; therefore, a recommendation bias might exist. The number of patients enrolled in this study was limited, and this study lacked long-term follow-up of this series of data on the fundus, such as macular/GCC/RNFL thickness, VF, and cpVD, which might result in further fundus changes to the PAACG patients. However, from our current research data, it was enough to show that for PAACG patients, the attack time and retinal changes were clearly correlated.

According to our results, cpVD had more obvious changes than RNFL thickness, macular thickness, and GCC thickness. We considered that OCT-A vessel indicators may be more sensitive than SD-OCT at the early stage of PAACG. Therefore, OCT-A is a promising technology for clinical monitoring of vascular changes in glaucoma. Its application may help the ophthalmologist further understand the pathophysiology of glaucoma and its underlying vascular mechanisms.

In summary, longer attack time indicated longer periods of high IOP and perfusion damage to the retinal vessels. It not only caused short-term damage to the fundus but also played a negative role in subsequent fundus change process. Therefore, it is importantly necessary to control the IOP in time once the PAACG patients had the relevant symptoms.

## Figures and Tables

**Table 1:** Characteristics of patients with acute primary angle closure.

Characteristic (*n* = 26)	Group A (*n* = 13)	Group B (*n* = 13)	*p* ^*∗*^
Age	63.92 ± 8.06 (52∼85)	69.46 ± 8.02 (57∼82)	0.092
Sex (male/female)	6/7	7/6	0.855
Right eye/left eye	7/6	8/5	0.877
Duration of symptoms (hours)	10.0 ± 6.2 (2∼19)	79.3 ± 24.7 (28∼120)	0.001
Cataract surgery (yes/no)	13/0	13/0	1.000
IOP preoperative (mmHg)	49.54 ± 12.59 (29∼78)	46.92 ± 9.68 (28∼62)	0.558
IOP postoperative (mmHg)	12.77 ± 2.65 (9∼17)	12.77 ± 3.85 (6∼19)	0.834
Mean cpRNFL thickness	108.32 ± 10.25	100.46 ± 12.75	0.327
Vertical cup-to-disc ratio	0.56 ± 0.13	0.68 ± 0.23	0.087
Mean GCC thickness	85.32 ± 6.57	80.25 ± 8.27	0.076
cpVD	0.59 ± 0.06	0.55 ± 0.05 3	0.06
MD	−2.57 ± 1.45	−3.85 ± 2.78	0.056

cpVD: circumpapillary retinal nerve fiber layer vessel density; MD: mean defect.

**Table 2:** Comparison of postoperative characteristics between group A and group B.

	Group A (*n* = 13)	Group B (*n* = 13)	*p* ^*∗*^
BCVA (logMAR)			
Baseline	1.56 ± 0.89	0.97 ± 0.64	0.058
Final	0.25 ± 0.15	0.46 ± 0.51	0.167
Improvement	1.32 ± 0.84	0.50 ± 0.21	0.004
VF			
MD value	−3.65 ± 2.54	−16.05 ± 5.99	<0.001
PSD value	2.75 ± 1.10	9.43 ± 2.93	<0.001
Macular thickness (*μ*m)			
Fovea area	255.00 ± 27.94	203.92 ± 59.73	0.010
Inner area	304.38 ± 23.41	254.23 ± 58.13	0.008
Outer area	269.92 ± 21.68	231.00 ± 32.39	0.001
GCC thickness (*μ*m)	82.62 ± 8.76	65.23 ± 18.56	0.005
RNFL thickness (*μ*m)			
Superior area	137.54 ± 12.23	103.92 ± 27.74	0.001
Temporal area	78.25 ± 11.93	75.15 ± 7.67	0.543
Inferior area	149.92 ± 21.73	100.38 ± 24.38	<0.001
Nasal area	68.08 ± 4.29	59.31 ± 14.67	0.057
Average area	105.08 ± 9.38	77.69 ± 20.23	<0.001
cpVD			
Central area	0.34 ± 0.03	0.29 ± 0.04	0.002
Inner area	0.56 ± 0.03	0.42 ± 0.06	<0.001
Outer area	0.56 ± 0.03	0.40 ± 0.08	<0.001
Full area	0.56 ± 0.02	0.41 ± 0.07	<0.001

BCVA: best-corrected visual acuity; logMAR = logarithm of the minimum angle of resolution. MD: mean defect; PSD: pattern standard deviation.

**Table 3:** Pearson correlation for acute primary angle closure glaucoma eye.

Variable	cpVD	VF MD	VF PSD
Mean RNFL thickness	*r* = 0.761, *p* < 0.001	*r* = 0.772, *p* < 0.001	*r* = −0.736, *p* < 0.001
Mean GCC thickness	*r* = 0.605, *p*=0.001	*r* = 0.605, *p*=0.001	*r* = −0.516, *p*=0.007
cpVD		*r* = 0.832, *p* < 0.001	*r* = −0.803, *p* < 0.001

## Data Availability

The clinical data used to support the findings of this study are available from the corresponding author upon request.
